# Comparative Proteomic Analysis of Polarized Human THP-1 and Mouse RAW264.7 Macrophages

**DOI:** 10.3389/fimmu.2021.700009

**Published:** 2021-06-29

**Authors:** Pengfei Li, Zhifang Hao, Jingyu Wu, Chen Ma, Yintai Xu, Jun Li, Rongxia Lan, Bojing Zhu, Pengyu Ren, Daidi Fan, Shisheng Sun

**Affiliations:** ^1^ College of Life Science, Northwest University, Xi’an, China; ^2^ Department of Neurosurgery, The Second Affiliated Hospital of Xi’an Jiaotong University, Xi’an, China; ^3^ Shaanxi Key Laboratory of Degradable Biomedical Materials, School of Chemical Engineering, Northwest University, Xi’an, China

**Keywords:** macrophage, polarization, cell model, proteomics, mass spectrometry

## Abstract

Macrophages can be polarized into classically activated macrophages (M1) and alternatively activated macrophages (M2) in the immune system, performing pro-inflammatory and anti-inflammatory functions, respectively. Human THP-1 and mouse RAW264.7 cell line models have been widely used in various macrophage-associated studies, while the similarities and differences in protein expression profiles between the two macrophage models are still largely unclear. In this study, the protein expression profiles of M1 and M2 phenotypes from both THP-1 and RAW264.7 macrophages were systematically investigated using mass spectrometry-based proteomics. By quantitatively analyzing more than 5,000 proteins among different types of macrophages (M0, M1 and M2) from both cell lines, we identified a list of proteins that were uniquely up-regulated in each macrophage type and further confirmed 43 proteins that were commonly up-regulated in M1 macrophages of both cell lines. These results revealed considerable divergences of each polarization type between THP-1 and RAW264.7 macrophages. Moreover, the mRNA and protein expression of CMPK2, RSAD2, DDX58, and DHX58 were strongly up-regulated in M1 macrophages for both macrophage models. These data can serve as important resources for further studies of macrophage-associated diseases in experimental pathology using human and mouse cell line models.

## Introduction

Macrophages are important immune cells, which participate in a series of inflammations and autoimmune diseases through either specific (cellular immunity) or non-specific defenses (innate immunity) *in vivo* (1). Macrophages have strong plasticity, heterogeneity and pluripotency. They can differentiate into different phenotypes and perform specialized functions in different micro-environments. According to the state of activation and function, macrophages can be mainly classified into classically activated macrophages (M1) and alternative activated macrophages (M2) (2, 3). M1 macrophages participate in the positive immune responses by secreting pro-inflammatory cytokines and chemokines and play a role in immune surveillance (4). M2 macrophages secrete anti-inflammatory cytokines, such as IL-4, IL-10 and TGF-β, and down-regulate immune responses to control immune regulation and tissue remodeling (5–7).

A balance among different activated states of macrophages is important for body health, and the imbalances of activation and inhibition of M1 and M2 phenotypes have been proved to be associated with the development of many diseases (8). It has been proposed that over-activation of M1 macrophages were related to pathogenic mechanisms of several inflammatory, autoimmune and chronic diseases (8, 9). The failure of inflammation subsidence may lead to chronic inflammatory autoimmune disease accompanied by irreversible tissue damage (10). Other researches have shown that M2 macrophages could promote tumor cell proliferation, invasion and angiogenesis in tumor micro-environment (11). These evidences indicated that macrophage polarization plays a key role in different diseases.

There has been much research concerned with macrophage polarization or macrophage-associated diseases through transcriptomic or proteomic approaches. It has been found that interferon-inducible proteins with tetrapeptide repeats (IFIT1, IFIT2, and IFIT3) are highly up-regulated in human and mouse M1 macrophages, and may serve as useful markers of atherosclerosis (12). However, there are restrictions and challenges that obstructed research progress. On the one hand, evidences revealed that many markers are not shared between human and mouse polarized macrophages. A study shows that alternatively activated myeloid cells in murine and human exhibit distinct differences at the transcriptome level, indicating Arginase-1 and Ym1 are markers for murine, but not for human (13). On the other hand, limited cell numbers, laborious preparation and genetic/epigenetic differences between donors make it difficult to do macrophage-associated researches based on primary cells (14).

Two classical cell lines human THP-1 and mouse RAW264.7 have been extensively used to study macrophage functions, mechanisms, and signaling pathways (14–16). THP-1 is a human myeloid leukemia mononuclear cell line, and RAW264.7 is a mouse leukemia cell line of monocyte macrophage. Advantages of using the two cell lines over primary macrophages are listed as follows: cell lines are relatively easy and safe to use; the growing rate of cell lines is much higher than that of primary cells; the homogeneous genetic background of cell lines could minimize the degree of variability in the macrophage phenotypes (17). Nonetheless, polarized macrophages are still very different between human and mouse cell lines. Insufficient information for cell models can lead to unrealistically experiment data or improper use of models. To make a better use of mouse or human cell model in the studies of macrophage-associated diseases, it is necessary to systematically comparative protein expression profiles and the related biological functions between human THP-1 and mouse RAW264.7 polarized macrophages.

In this study, due to the fact that human THP-1 and mouse RAW264.7 cell lines have different tolerances and responses to the stimuli, we selected the classic polarization methods for each cell line to obtain classical activated M1 and alternatively activated M2 macrophage phenotypes. Then, we comprehensively identified and quantified proteins in different types of macrophages from both cell lines through high-throughput proteomics. In addition, some key proteins were further validated using parallel reaction monitoring (PRM) analysis and qPCR. These proteins that were commonly or uniquely expressed in each type of polarized macrophages in human THP-1 and mouse RAW264.7 macrophage models provide available references for further studying the mechanisms of immune regulation.

## Materials and Methods

### Cell Culture and Stimulation

Human monocytic cell line (THP-1) and mouse monocytic cell line (RAW264.7) were obtained from the cell bank of Chinese Academy of Sciences (Shanghai, China). The cell lines were tested for mycoplasma contamination before being used in our experiments. THP-1 monocytes were cultured in RPMI 1640 medium (HyClone, USA), supplemented with 10% fetal bovine serum (FBS; Biolnd, Israel) and 1% penicillin–streptomycin (Solarbio, China) at 37°C and 5% CO_2_. RAW264.7 monocytes were cultured in DMEM medium (HyClone, USA), supplemented with 10% FBS and 1% penicillin–streptomycin at 37°C and 5% CO_2_. The THP-1 monocytes were differentiated to macrophages with 10 ng/ml phorbol-12-myristate-13-acetate (PMA) for 24 h. The THP-1 macrophages were subsequently stimulated with human IFN-γ (50 ng/ml) and LPS (15 ng/ml) for 48 h to M1 phenotype, or stimulated with human IL-4 (25 ng/ml) and IL-13 (25 ng/ml) for 72 h to M2 phenotype. As for RAW264.7 macrophages, the cells were stimulated by mouse IFN-γ (2.5 ng/ml) and LPS (200 ng/ml) for 24 h to M1 phenotype, or by mouse IL-4 (10 ng/ml) for 48 h to M2 phenotype. The untreated RAW264.7 cells and PMA-THP-1s were used as M0 phenotype. PMA, LPS, IFN-γ, IL-4 and IL-13 were all purchased from Beyotime (Shanghai, China).

### Immunofluorescence

Cells were polarized by different stimulus as described above. After three times of wash by PBS buffer, cells were fixed in 4% paraformaldehyde (Solarbio, Beijing, China) at room temperature for 30 min, and then permeabilized with 0.25% Triton X-100 (Solarbio, Beijing, China) for 5 min (only for protein iNOS). Nonspecific binding of the antibodies was blocked by adding 5% BSA (Solarbio, Beijing, China) at room temperature for 1 h. The rabbit polyclonal antibody against iNOS (GB11119, Servicebio, Wuhan, China), MHC II (bs-8481R), CD163 (bs-2527R) and CD206 (bs-21473R, Bioss, Beijing, China) was used to incubate cells with the ratio of 1:500 (antibody to PBS, v/v) in a humidified chamber overnight at 4°C, respectively. The cells were incubated with the secondary antibodies. Cy3 conjugated Goat anti-rabbit antibody (GB21303, Servicebio, Wuhan, China) was used to combine the primary antibody against iNOS and MHC II at 1:400 dilution for 2 h at room temperature in the dark. Goat anti-rabbit Alexa Fluor^®^ 488 secondary antibody (GB25303, Servicebio, Wuhan, China) was used to combine the primary antibody against CD163 and CD206 at 1:400 dilution for 2 h at room temperature in the dark. Then nuclei were stained with DAPI (Solarbio, Beijing, China) for 10 min. The cells were dropped of sealing agent (Servicebio, Wuhan, China) which was against fluorescence quenching. Images were acquired on an inverted fluorescence microscope (CKX53, Olympus, Japan) and analyzed using its own MShot Image analysis system.

### Cell Lysis and Protein Extraction

The details of sample treatment were described previously (18). Treated and untreated cells were washed three times by ice-cold PBS buffer to remove the cell culture medium. The denaturing buffer containing 8 M urea and 1 M ammonium bicarbonate was added to each cell culture dish for cell lysis. Cell lysate of each sample was sonicated by ultrasonic cell distribution system at 60% power for 10 min (10 s break after each 8 s sonication) in an ice bath until the solution became clear. The samples were then centrifuged at 14,000*g* for 15 min at 4°C and the supernatants were collected. The protein concentration was measured by BCA protein assays (Beyotime, Shanghai, China). Cellular proteins were harvested from three biological replicates at each condition, and the proteins were pooled into one sample for further sample preparation.

### Protein Digestion and Peptide Desalting

Denatured proteins in denaturing buffer were reduced by 5 mM dithiothreitol (DTT) at 37°C for 1 h and then alkylated by 15 mM iodoacetamide (IAM) at room temperature in the dark for 30 min. Reaction was terminated by additional 2.5 mM DTT at room temperature for 10 min. The solution was diluted two times with ultra-pure water, and then the proteins were digested by sequencing grade trypsin (Promega, Madison, WI, USA) with the ratio of 1:100 (trypsin to total protein, w/w) at 37°C for 2 h. The solution was further diluted four times with ultra-pure water and additional trypsin (trypsin to total protein, 1:100, w/w) was added with overnight incubation at 37°C overnight. After protein digestion, the pH of the solution was adjusted with 10% trifluoroacetic acid (TFA) till pH <2. The sample solution was centrifuged at 15,000*g* for 10 min and the peptides in the supernatant were desalted using hydrophile–lipophile balance (HLB) columns (Waters, Milford, MA, USA). The peptides were eluted from the column by 60% acetonitrile (ACN)/0.1% TFA, dried by SpeedVac and resuspended in 20 μl of 0.1% formic acid (FA) solution for LC–MS/MS analysis.

### LC–MS/MS Analysis

Each peptide sample underwent triplicate LC−MS/MS runs using an Orbitrap Fusion Lumos Mass Spectrometer (Thermo Fisher Scientific, Germany) coupled with an online EASY-nanoLC™ 1200 instrument (Thermo Fisher Scientific, Germany). Samples were first loaded onto a 75 μm × 2 cm nanoViper PepMap™100 C18 precolumn and then separated on a 75 μm × 50 cm nanoViper PepMap™100 C18 analytical column (Thermo Fisher Scientific, Germany). Mobile phase consisted of 0.1% FA (A) and 0.1% FA/80% ACN (B). The gradient profile (240 min) was set as follows: 3–7% B for 2 min, 7–35% B for 166 min, 35–68% B for 40 min, 68–99% B for 10 min, and 99% B for 22 min. The parameters of mass spectrometry were set as follows: for MS1, scan range of orbitrap spectra (automatic gain control AGC 4 × 10^5^) were from 350 to 1,800 m/z at a resolution of 60 K. For MS2, the multiply charged ions were fragmented in the collision cell by higher-energy collisional dissociation (HCD, collision energy 30%) with an isolation window of 1.6 m/z, a maximum injection time of 30 ms, a resolution of 15 K and AGC target of 5 × 10^4^.

### Global Database Search and Protein Quantification

All generated raw files were submitted to Proteome Discover (PD, version 2.3, Thermo Fisher Scientific, Germany) with label-free quantitation (LFQ) analysis. The human and mouse protein sequence databases were downloaded from UniProt database in May 2019 (http://www.uniprot.org). Database searching was performed with the following parameters: cysteine carbamidomethylating (C, +57.0215 Da) as a fixed modification; methionine oxidation (M, +15.9949 Da) and N-terminal acetylation (+42.010565 Da) as variable modifications; up to two missed cleavage sites were permitted for trypsin digestion; the tolerances of precursor and fragment masses were set at 10 ppm and 0.02 Da, respectively; 1% protein false discovery rate (FDR) was used as the filter for both protein and peptide identification, and at least two peptide-spectrum matches (PSMs) were required for a peptide identification. Label-free method in PD was used for relative quantification of proteins among samples. LFQ was performed for calculation of protein abundances. Protein ratios were calculated as the median of all pairwise ratios calculated between the three replicates of all peptide abundances.

### Bioinformatics Analysis

Gene Ontology (GO) enrichment and Kyoto Encyclopedia of Genes and Genomes (KEGG) pathway analyses of the significantly different proteins between M1/M2 and M0 (control) macrophages was performed using DAVID software (https://david.ncifcrf.gov) (19) and ClueGO plug-in and Cluepedia of Cytoscape software (20). The results were filtered with the thresholds of count >2 and *P*-value <0.05. Protein interactions were analyzed using STRING database (https://string-db.org) (21) and the interactions with a combined score >0.4 were selected to construct the PPI networks using the Cytoscape software. To screen core proteins, the MCODE plugin for Cytoscape was used to identified highly interconnected clusters in the PPI network. Principal component analysis (PCA) was performed on three phenotypes (M0, M1, and M2) from human THP-1 and mouse RAW264.7 cells based on the abundance of quantitative proteins by using the “gmodels” package in R language. Hierarchical clustering analysis of the differential abundance proteins was conducted by the “pheatmap” R package. The data were first Z-score normalized with matrix access by rows and then clustered using the Pearson correlation for distance calculation and average for clustering method.

### Parallel Reaction Monitoring

Based on the global proteome identification and PD data analysis as described above, a spectral library was built in Skyline 20.1 (22) and target unique peptides from initial quantitative proteins were selected. The mass list table of all precursor ions incorporated peptide sequence, mass-to-charge ratio (m/z), charge state, and elution time. PRM experiments were performed on a LC/MS-MS system in PRM mode with an isolation width of 0.7 m/z, a maximum injection time of 100 ms, and the HCD collision energy of 30%. All PRM-MS raw files were processed in Skyline and the sum of the peak area for each protein was generated.

### RNA Extraction and qPCR

Total RNA was isolated from cells using a kit (Sangon Biotech, Shanghai, China) and was reverse-transcribed by using high-capacity cDNA reverse transcriptase kit (TaKaRa, Japan). qPCR assays were performed with SYBR Green PCR Master Mix (TaKaRa, Japan) and a Fast qPCR System (Applied Biosystems, USA). Gene specific primers were designed and purchased from GeneCreate (Wuhan, China). The sequence of primers was shown in **Table S1**. The glyceraldehyde-3-phosphate dehydrogenase (GAPDH) was selected as internal controls. Three biological replicates were used for each sample. Gene expression was normalized to internal controls and quantified relative to its expression in M0 cells using the 2^−ΔΔCt^ method. The data were subjected to the Student’s t-test and difference was considered significant with *P <*0.05.

## Results

### Polarization of Human THP-1 and Mouse RAW264.7 Cell Lines Into M1 and M2 Macrophages

**Figure 1:**
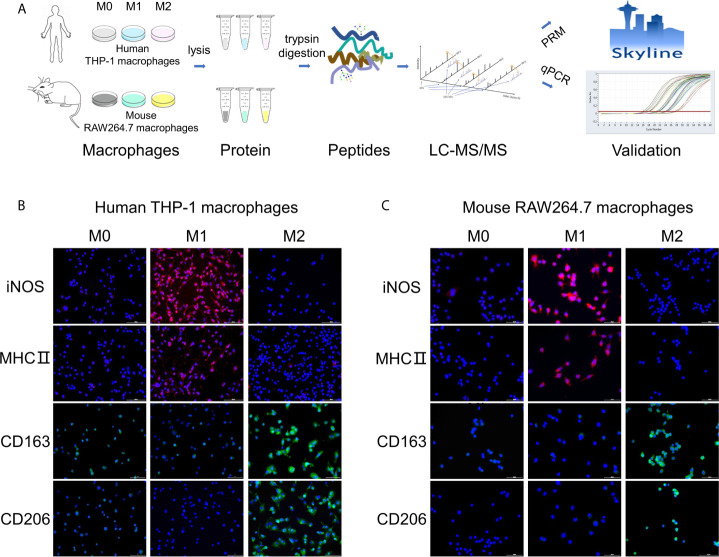
Workflow of the study and validation of polarization models. **(A)** Workflow of this study about quantitative proteome analysis of three types of polarized macrophages from human THP-1 and mouse RAW264.7 cells. **(B, C)** Measurement of known markers for M1/M2 phenotypes in polarized human THP-1 macrophages **(B)** and mouse RAW264.7 macrophages **(C)** using the immunofluorescence staining method. Red: M1 macrophage markers iNOS and MHC II; Green: M2 macrophage markers CD163 and CD206; Blue: DAPI (nucleated cells) in untreated or differently treated cells. Scale bar = 10 μm.

In this study, differently polarized macrophages of two cell lines were analyzed by quantitative proteomics (**Figure 1A**). For human macrophages, THP-1 monocytes were differentiated to M0 macrophages by PMA. M0 macrophages were polarized into M1 with human IFN-γ and LPS, into M2 with human IL-4 and IL-13 as described before (23). For mouse macrophages, untreated RAW264.7 cells were used as M0 macrophages. M0 macrophages were polarized into M1 with mouse IFN-γ and LPS, into M2 with mouse IL-4, according to previous methods (24). After polarization, different types of macrophages showed significantly different morphology through the microscope (**Figure S1**).

To evaluate the model of M1/M2 macrophages, the known markers of M1/M2 phenotypes for THP-1 and RAW264.7 macrophages were measured by using the immunofluorescence approach. Two common markers of M1-polarized macrophages for THP-1 and RAW264.7 cell lines, iNOS and MHC II proteins (25, 26), were marked with red fluorescent and significantly increased in M1 phenotypes in comparison to M0 and M2 phenotypes (**Figures 1B, C**). Similarly, two common M2 markers, CD163 and CD206 (27, 28), were marked with green fluorescent and remarkably increased in M2 phenotypes compared with M0 and M1 (**Figures 1B, C**). These results confirm the successful polarization of M0 to M1 or M2 by using the above polarization methods.

To further confirm the polarized M1/M2 macrophages, we checked more markers or over-expressed proteins that have been reported previously by using our proteomic data to supplement immunofluorescence data (**Figure S2**). More than twenty markers of M1 and M2 phenotypes from two cell lines were identified from the proteome data. In THP-1 macrophages, these significantly up-regulated proteins in M1 type included SOD2 (12.8-fold) (29), OASL (11.9-fold), CD40 (5.1-fold), and NFκB2 (2.7-fold) (12); while M2 markers included TGM2 (28-fold) (30) and CD209 (2.3-fold) (31). In addition, the over-expression of LSP1 (5-fold) was also detected in M2 type, which is consistent with a previous study (12). In RAW264.7 macrophages, the M1 markers NOS2 (iNOS, 36.9-fold) and CD86 (8.6-fold) (32), as well as the M2 marker MRC1 (CD206, >100-fold) were also dramatically increased in the related macrophages. In addition, PCA using the quantitative proteins from either human THP-1 or mouse RAW264.7 macrophages resulted in a clear separation of three groups, representing M0, M1 and M2 phenotype, respectively (**Figures S3A, B**). These results further confirm the credibility of polarization of M0 macrophages to M1/M2 phenotypes for both cell lines.

### Differentially Expressed Proteins Among Three Subtypes of Human THP-1 Macrophages

**Figure 2:**
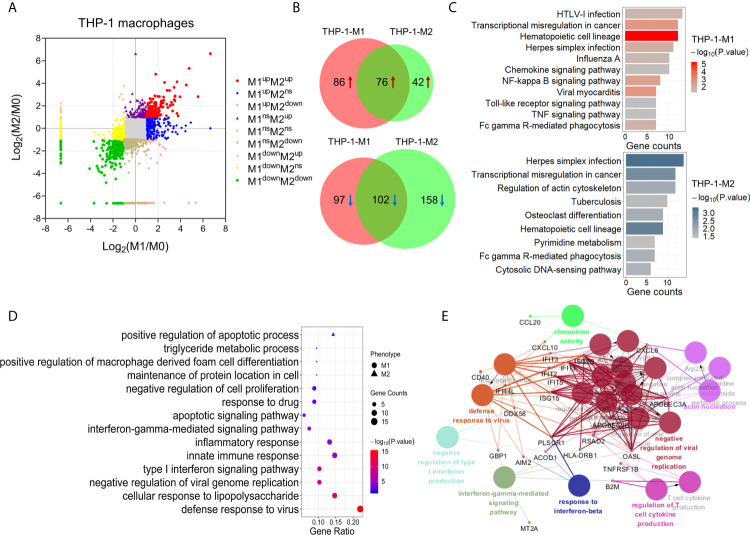
Identification and bioinformatic analysis of differentially expressed proteins from M1 and M2 types of polarized human THP-1 macrophages. **(A)** Scatter plot showing the distribution of differentially expressed proteins in M1 and M2 polarized macrophages, compared with the M0 macrophages. **(B)** Venn diagram of differentially expressed proteins between two types of polarized cells. Upper: up-regulated; lower: down-regulated. **(C)** KEGG pathway analysis of differentially expressed proteins in M1 (upper) and M2 (lower) macrophages from human THP-1 cells. **(D)** Gene oncology enrichment analysis (biological process) of up-regulated proteins in M1 or M2 phenotype. **(E)** The enriched biological processes corresponding to the up-regulated proteins in M1 macrophages (*P*-value <0.05).

We first investigated the protein expression differences among three subtypes of human THP-1 macrophages using the label-free quantitative proteomics. Among 6,682 protein that were identified from THP-1 macrophages with a FDR cutoff of 0.01, and 5,136 were quantitative among samples (**Figure 2A** and **Table S2**). To ensure the accuracy and reliability of our results, proteins that had at least five PSMs and 4-fold changes in the M1 or M2 macrophages compared with M0 was considered as significantly expressed proteins. Using this cutoff, a total of 361 proteins were significantly changed in M1 macrophages (compared with M0), including 162 up-regulated and 199 down-regulated (**Table S3**). In polarized M2 macrophages, there were 378 significantly changed proteins (compared with M0), of which 118 were up-regulated and another 260 were down-regulated (**Table S4**). Among these significantly changed proteins, 76 up-regulated and 102 down-regulated proteins were in common between M1 and M2 macrophages (**Figure 2B** and **Table S5**).

KEGG pathway analysis showed that the proteins significantly changed in M1 macrophages were mainly involved in the pathways of transcriptional misregulation in cancer, NF-kappa B signaling pathway, TNF signaling pathway, and viral myocarditis. While the proteins significantly changed in M2 macrophages were mainly involved in the pathways of herpes simplex infection, regulation of actin cytoskeleton, tuberculosis, osteoclast differentiation, and pyrimidine metabolism (**Figure 2C**).

We then focused on the proteins that were uniquely up-regulated in one subtype of polarized THP-1 macrophages. Compared with M0 and M2, 68 proteins were uniquely up-regulated in M1 macrophages (**Table S6**). Gene ontology analysis showed that these proteins were mainly involved in the biological processes of the defense response to virus, cellular response to lipopolysaccharide (LPS), innate immune response, inflammatory response, type I interferon signaling pathway, and interferon-gamma-mediated signaling pathway, etc. (**Figures 2D, E**). These results were consistent with those expected from the M1 pro-inflammatory effects. Similarly, there were 21 proteins uniquely up-regulated in M2 compared with M0 and M1 macrophages (**Table S7**). These M2 specific proteins were mainly enriched (*P <*0.05) in the biological processes of positive regulation of macrophage derived foam cell differentiation, triglyceride metabolic process, and positive regulation of apoptotic process (**Figure 2D**).

Among proteins that were uniquely up-regulated in M1 macrophages, seven proteins including IFIT1, IFIT2, IFIT3, CD14, CD38, CD40, and CXCL10 had been reported as M1 biomarkers or cytokines/chemokines in previous studies (33–35). Similarly, the uniquely up-regulated protein in M2 macrophage, TGM2, had also been used as a M2 biomarker (30). These results further confirmed the good quality of our proteomic data and the reliably polarized macrophage models for this study. Moreover, according to our results and further analysis as described above, many other uniquely up-regulated proteins in specific types of polarized macrophages exhibited a high degree of interaction and participated in important biological process. These included USP18, CD274, RSAD2, IDO1, GBP1, DDX58, and TAP1 for M1 macrophages, and GPC4, DBN1, LPL, SCAMP1, and NVL for M2 macrophages (**Table S8**).

### Differentially Expressed Proteins Among Three Subtypes of Mouse RAW264.7 Macrophages

**Figure 3:**
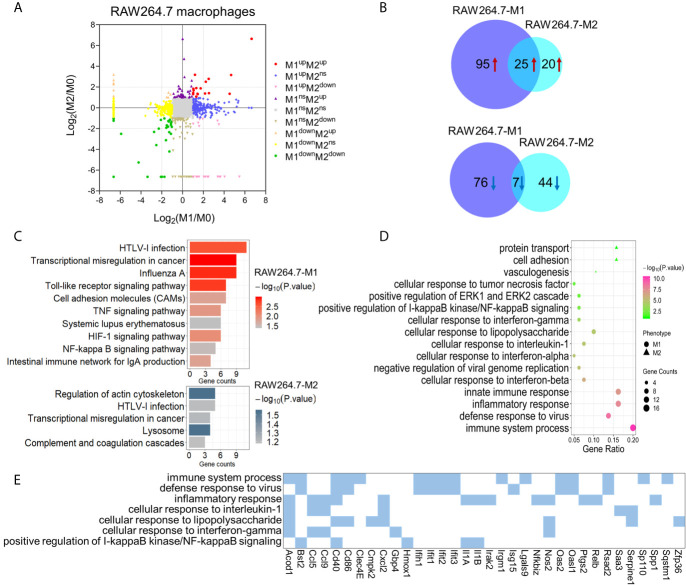
Identification and bioinformatic analysis of differentially expressed proteins from M1 and M2 types of polarized mouse RAW264.7 macrophages. **(A)** Scatter plot showing the distribution of differentially expressed proteins in M1 and M2 polarized macrophages, compared to the M0 macrophages. **(B)** Venn diagram of differentially expressed proteins between two types of polarized macrophages. Upper: up-regulated; lower: down-regulated. **(C)** KEGG pathway analysis of differentially expressed proteins in M1 (upper) and M2 (lower) macrophages from mouse RAW264.7 cells. **(D)** Gene ontology enrichment analysis (biological process) of up-regulated proteins in M1 and/or M2 phenotypes. **(E)** The enriched biological processes corresponding to the up-regulated proteins in M1 macrophages.

Using the same global LFQ workflow as for the PMA-THP-1 cellular proteins above, we identified 6,268 proteins from the RAW264.7 macrophages, of which 5,188 were quantitative among three subtypes (**Figure 3A** and **Table S9**). Compared with M0 macrophages, 203 proteins were identified to be significantly changed (4-fold, PSM ≥5) in M1 macrophages, of which 120 were up-regulated and other 83 were down-regulated (**Table S10**). Meanwhile, there were 96 proteins that had significant alterations in M2 macrophages, including 45 up-regulated and 51 down-regulated (**Table S11**). Among these, 25 proteins were commonly up-regulated and seven were commonly down-regulated in both M1 and M2 macrophages (**Figure 3B** and **Table S12**). KEGG pathway analysis showed that the differentially expressed proteins in M1 phenotype were mainly enriched in pathways related to Toll-like receptor signaling pathway, TNF signaling pathway, and NF-kappa B signaling pathway. The differentially expressed proteins in M2 phenotype were mainly involved in regulation of actin cytoskeleton, and HTLV-I infection (**Figure 3C**).

We then focused on the proteins that were uniquely up-regulated in one subtype of polarized RAW264.7 macrophages. Among these differently expressed proteins, 87 proteins were significantly up-regulated in M1 cells compared with M0 and M2 cells, and 19 proteins were significantly up-regulated in M2 cells compared with M0 and M1 macrophages (**Table S13**). Gene ontology analysis showed that the proteins uniquely up-regulated in M1 macrophages were mainly involved in innate immune response, defense response to virus, cellular response to lipopolysaccharide, and cellular response to interferon-gamma (**Figures 3D, E**). While the proteins uniquely up-regulated in M2 macrophages were mainly enriched in protein transport, cell adhesion, and vasculogenesis (**Figure 3D**). These pathways also reflected the distinct functions of M1 and M2 macrophages. Among these proteins that were uniquely up-regulated in M1 macrophages, NOS2, CD40, and CD86 have been used as M1 macrophage markers in previous studies (36). Based on our results, a number of other proteins may also play an important role in the polarization process for mouse macrophages, include ACOD1, GBP4, PLAUR, CMPK2, and PTGS2 for M1, as well as STAT5B, EPHA2, CLEC10A, and CASP6 for M2 (**Table S14**).

### Similarities of Protein Expression Between Human THP-1 and Mouse RAW264.7 Polarized Macrophages

**Figure 4:**
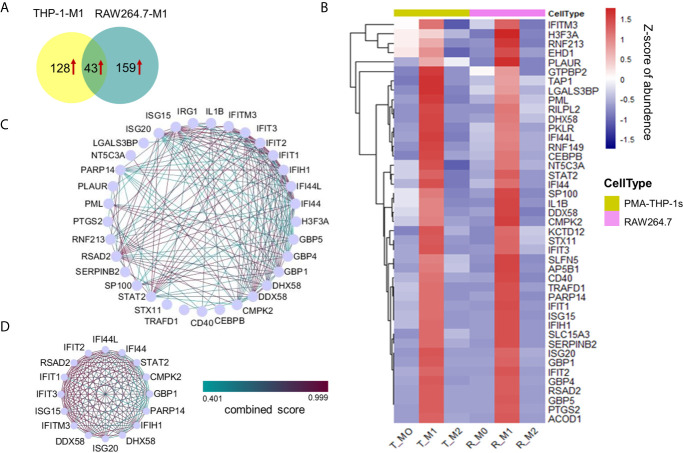
Proteins uniquely up-regulated in M1 macrophages from both the human THP-1 and mouse RAW264.7 cell lines. **(A)** Venn diagram showing 43 uniquely up-regulated proteins in M1 macrophages for both human THP-1 and mouse RAW264.7 cells. **(B)** Cluster analysis of 43 up-regulated proteins in M1 macrophages with z-scored of protein abundance among three phenotypes from two cell lines. **(C)** PPI network of highly interconnected proteins among 43 up-regulated proteins in M1 macrophages. **(D)** A module was extracted by MCODE with score = 15.333.

Based on the results above, only 13 unique proteins were commonly up-regulated in M1 macrophages of human THP-1 and mouse RAW264.7 cell lines (**Table S15**). Considering the high homology between mouse and human genes and their regulatory sequences, the cutoff of protein expression difference in M1 phenotype was adjusted to a 2-fold change (a comparison with M0 and M2 cells). Among 171 (and 202 proteins) that were uniquely up-regulated in M1 polarized THP-1 (and RAW264.7) macrophages, 43 of which were commonly up-regulated in M1 macrophages of both THP-1 and RAW264.7 cell lines (**Figure 4A** and **Table S16**). Hierarchical clustering based on the abundances of these proteins within three phenotypes of two cell lines clearly showed that these proteins were highly expression at the M1 compared with M0 and M2 macrophages (**Figure 4B**). Nevertheless, no commonly up-regulated protein (2-fold change) was identified in M2 macrophages of both THP-1 and RAW264.7 cell lines (a comparison with M0 and M1 macrophages). When adjusting the cutoff to a 1.5-fold change, we identified five commonly up-regulated proteins in M2 macrophages of both cell lines, including MYO6, NDRG1, LSP1, CD81, and GM2A.

The protein-protein interactions existed in 33 of 43 proteins (**Figure 4C**). A large part of these interacting proteins was involved in the biological process of defense response or defense response to virus, including seven interferon-induced proteins: IFIT1, IFIT2, IFIT3, IFIH1, IFITM3, IFI44, and IFI44L; three guanylate-binding proteins: GBP1, GBP4, and GBP5; two interferon-stimulated gene products: ISG20, and ISG15; two probable ATP-dependent RNA helicase: DDX58, and DHX58; etc. Among these M1-specific proteins, five are known as M1 markers for mouse and/or human M1 macrophages, including IFIT1, IFIT2, IFIT3, IL1B (12), and CD40 (33). Moreover, ISG15 is a ubiquitin-like protein which plays a key role in the innate immune response to viral infection *via* its conjugation to a target protein (ISGylation) (37). STAT2 and CEBPB are two key transcription factors of macrophage polarization (38, 39). SerpinB2 is a member of the clade B that can inhibit catalytic activity of urokinase-type plasminogen activator and have important physiological functions during inflammation (40). A module containing 16 highly interconnected proteins were further identified by MCODE (**Figure 4D**). These core proteins may play a crucial role in M1 macrophages for both human and mouse species.

### Differences of Protein Expression Between Human THP-1 and Mouse RAW264.7 Polarized Macrophages

**Figure 5:**
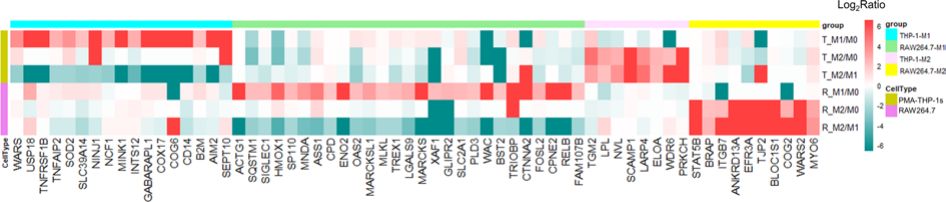
Differentially expressed proteins between human and mouse polarized macrophage models. There were 17 and 27 proteins uniquely up-regulated in M1 subtype of human THP-1 and mouse RAW264.7 cell line, respectively. Eight and 10 proteins were uniquely up-regulated in M2 subtype of human THP-1 and mouse RAW264.7 cell models, respectively.

Based on our results described above, a large number of significantly different proteins were only expressed in human THP-1 or mouse RAW264.7 macrophages, regardless of M1 or M2 polarization. To define the differences of protein expression of polarized macrophages between two cell lines, in our analysis, proteins uniquely identified or exclusively up-regulated (at least 2-fold increase with ≥5 PSMs) in the same type of either human THP-1 or mouse RAW264.7 polarized macrophages were considered as the differently expressed proteins between the two cell models (**Figure 5** and **Table S17**). These included 17 and eight uniquely up-regulated proteins in M1 and M2 types of human THP-1 macrophages, as well as 27 and 10 uniquely increased proteins in M1 and M2 types of mouse RAW264.7 macrophages, respectively. Among these, proteins over-expressed in M1 type of human THP-1 macrophages included several enzymes, such as WARS, USP18, SOD2, MINK1, and SEPT10, as well as the receptors TNFRSF1B and CD14. Differences in these over-expressed proteins between polarized THP-1 and RAW264.7 macrophages may be due to the differences of the two cell lines response to the same stimulus. To a certain extent, this study illustrated the differences of protein expression between human THP-1 and mouse RAW264.7 polarized macrophages, and it should be taken into consideration when studying macrophage-associated diseases using mouse cell models.

### Validation of Up-Regulated Proteins by Parallel Reaction Monitoring

To verify the high protein expression in M1 polarization of both human THP-1 and mouse RAW264.7 macrophages from the proteomics results, 16 of 43 up-regulated protein were arbitrarily selected for targeted quantitation by PRM. It has been proved that PRM is more accurate and reproducible compared with the relative quantification by western blot and immunofluorescence (41). Results showed all proteins were up-regulated in M1 cells, which was consistent with the global proteomics data (**Table 1**). This confirmed the changes observed by global LFQ proteomics methods.

**Table 1:** Commonly up-regulated proteins changes of M1 polarization in both the THP-1 and RAW264.7 cell models determined by global proteomics and PRM.

Gene Name	Global Proteomics fold change				PRM fold change			
	PMA-THP-1s		RAW264.7		PMA-THP-1s		RAW264.7	
	M1/M0	M2/M0	M1/M0	M2/M0	M1/M0	M2/M0	M1/M0	M2/M0
CMPK2	4.27	0.37	20.94	0.72	5.99	0.30	16.38	0.55
RSAD2	10.29	1.00	4.91	0.68	65.89	0.96	304.22	2.09
DDX58	6.17	0.57	3.05	0.83	5.02	0.37	2.79	0.73
DHX58	2.38	0.68	3.17	0.90	12.93	0.03	2.06	0.96
CEBPB	4.12	1.29	3.13	0.70	6.56	2.09	10.71	0.35
GBP4	5.54	0.65	10.06	0.87	30.10	0.23	38.59	0.35
IFI44L	5.87	0.01	3.50	0.63	46.26	2.01	2.54	0.48
IFIT1	7.43	0.57	12.91	0.94	5.90	0.15	292.01	0.45
IL1B	2.56	0.15	5.48	1.28	6.17	0.03	163.97	2.75
LGALS3BP	2.12	0.55	2.37	0.63	7.65	0.76	2.41	0.46
NT5C3A	3.58	0.55	6.21	0.99	4.02	0.78	2.30	0.73
PLAUR	4.50	2.25	32.36	0.74	4.35	1.93	18.74	0.62
PML	3.78	0.67	2.44	1.03	4.39	0.82	2.32	0.74
PTGS2	100.00	100.00	30.77	0.78	413.83	53.66	150.38	1.39
SLC15A3	100.00	100.00	8.90	0.91	2.91	1.71	229.08	5.01
SLFN5	3.52	1.89	6.96	1.07	4.66	1.69	4.72	0.64

### Validation of Core Proteins at the Level of mRNA Transcription

**Figure 6:**
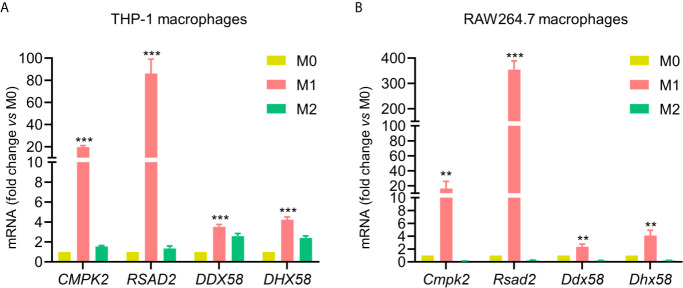
Validation of commonly up-regulated proteins in M1 for both cell lines. Validation of CMPK2, RSAD2, DDX58, and DHX58 mRNA expression in M0, M1 and M2 phenotypes from human THP-1 **(A)** and mouse RAW264.7 macrophages **(B)** by qPCR. Expression relative to M0 macrophages. All data are presented as mean ± SEM, n = 3. **P < 0.01, ***P < 0.001, compared with M0 control.

Based on quantitative proteomics results combined with bioinformatics analysis, CMPK2, RSAD2, DDX58, and DHX58 were observed as several core proteins among commonly up-regulated proteins under M1 conditions. To further verify proteomic analysis, we observed the mRNA expression levels of these four genes using qPCR. The results showed that mRNA level of four genes was consistent with the trends of their proteomics data. For both human THP-1 and mouse RAW264.7 macrophages, the four genes were significantly increased in M1 phenotype compared to M0 and M2 phenotypes (**Figures 6A, B**). To sum up, the mRNA and protein expression levels of these four genes increased significantly in response to IFN-γ and LPS stimulation for both macrophages.

### Comparison of Transcriptional and Translational Levels of Core Proteins in M0 Macrophages Between the Two Cell Models

Based on quantitative proteomics combined with qPCR, we compared transcriptional and translational levels of CMPK2, RSAD2, DDX58, and DHX58 in M0 macrophages between the two cell models (**Table 2**). Values were normalized to sample abundance of equivalent amounts, and ratios were calculated as the median of three independent experiments. The results showed that CMPK2, RSAD2, and DDX58 have higher (DHX58 has lower) transcriptional and translational levels in PMA-THP-1s than RAW264.7 macrophages.

**Table 2:** Comparison of transcriptional and translational levels of CMPK2, RSAD2, DDX58 and DHX58 in M0 macrophages between the two cell models.

Gene Name	PMA-THP-1s/RAW264.7	
	Ratio of mRNA levels	Ratio of protein levels
CMPK2	24.68	2.34
RSAD2	3.90	5.81
DDX58	9.55	1.60
DHX58	0.26	0.04

## Discussion

Macrophage activation is a dynamic and complex process that has beneficial or pathogenic effects to human health (42). Especially, uncontrolled activation of macrophages can lead to pathological diseases (43). During the immune response of the body, both rapid initiation and timely termination of the immune response are equally important in the defense of normal hosts against pathogen infection (44). Two polarized states of macrophages (M1 and M2) play important roles in pro-inflammatory and anti-inflammatory effects, respectively. Cell line models have been widely used for macrophage- associated research, therefore fully understanding protein expression characteristics of polarized macrophages from cell line models of different species base on comparative proteomics analysis is of great significance for the studies of polarization mechanism and preclinical development of therapies of inflammatory diseases.

In the study, we performed quantitative proteome analysis on differently polarized (and nonpolarized) macrophages from human THP-1 and mouse RAW264.7 cell lines. The PCA analysis above indicated that there were substantial differences in protein expression between M1 and M2 polarization in both cell lines. Based on differentially expressed proteins in the polarized macrophages, we identified some specific proteins whose expression is significantly enhanced under either M1 or M2 conditions. Bioinformatic analysis showed that the two subtypes perform different functions in the immune response. These up-regulated proteins in M1 were mainly involved in toll-like receptor signaling pathway, interferon-gamma-mediated signaling pathway, and defense response to virus, which are consistent with the pro-inflammatory function of M1 macrophages. Within the toll-like receptor signaling pathway, toll-like receptor 4 (TLR4) can recognize LPS, then activates intracellular signaling cascades to produce pro-inflammatory cytokines and chemokines, thereby initiates an immune response to clear pathogens (45). In the interferon-gamma-mediated signaling pathway, IFN-γ signals activate the STAT pathways, modulate interferon-stimulated genes (ISGs) expression, and participate in defense response (46). In contrast, M2 macrophages had anti-inflammatory activity and their up-regulated proteins identified in this study were mainly involved in cell adhesion, which may be related to phagocytosis, migration, chemotaxis, and tissue remodeling. Interestingly, both M2 macrophages of human and mouse were related to regulation of actin cytoskeleton. This result is compatible with the observed morphological differences between M1 and M2 macrophages that M2 shows a more elongated shape compared to M1 subtype. In addition, macrophage activation is closely connected to metabolic coordination (47). The first proteomic study on human M1 and M2 macrophages showed that the anaerobic glycolytic pathway is active in M1 macrophages, whereas the oxidative glucose metabolism and the fatty acid oxidation are mainly active in M2 macrophages (48). In our results, human THP-1 cells-derived M2 macrophages were mainly related to triglyceride metabolic process, and this process mainly involves oxidative glucose metabolism and the fatty acid oxidation. These evidences show substantial differences in metabolic routes for human M1 and M2 macrophages.

Our results also showed that the same type of polarized macrophages plays a very similar immune function in human THP-1 and mouse RAW264.7 cell models. Among 43 proteins that were commonly up-regulated in M1 phenotype of both cell models, some are known biomarkers, overexpressed proteins as well as transcription factors that regulate the polarization or proinflammatory activity in M1 macrophages, as stated in the Results section. For example, previous studies have shown that IFITs (IFIT1, IFIT2, and IFIT3) were strongly upregulated in M1 polarized human primary macrophages and IFIT1 can serve as a useful marker (in combination with other proteins) of M1 macrophages in mouse or in human pathology applications (12). In addition, our results show that SerpinB2 was strongly increased in M1 macrophages of two cell models. It has been suggested that SerpinB2 is often inducible under pro-inflammatory conditions, and it is one of the most upregulated proteins in macrophages and can represent >0.25% of total protein (40). These evidences demonstrate the reliability of our study. Importantly, the key findings of this study are that both the mRNA and protein levels of CMPK2, RSAD2, DDX58, and DHX58 were significantly up-regulated in M1 macrophages of both cell lines. The expression of CMPK2 (UMP-CMP kinase 2) in mitochondria will increase after macrophage sensing a foreign molecular cue, thereby driving an increase in the levels of the nucleotide cytidine triphosphate (CTP) which participate in synthesis of mitochondrial DNA. The freshly generated DNA is oxidized by reactive oxygen species (ROS), and then activates inflammasome, thereby producing inflammatory proteins that play a role in defense responses (49, 50). RSAD2 is induced by type I and type II interferon and can inhibit a wide range of DNA and RNA viruses (51). It has been reported that the RSAD2 has a catalytic effect, and then promotes the conversion of cytidine triphosphate (CTP) into a slightly different molecule ddhCTP that can be easily inserted into the viral genome instead of CTP, thereby preventing viral genome from adding new nucleotide element and terminates the replication process (52). DDX58 and DHX58 are members of the retinoic acid-inducible gene (RIG)-I-like receptors (RLRs) family that are a type of pattern-recognition receptors (PRRs) and can trigger innate immune responses against viral infections (53). IFN-γ significantly enhanced the DDX58 expression in THP-1 macrophages (54), and DDX58 was increased in M1 macrophages (compared to M0) and decreased in M2 macrophages for transcriptional signature of murine (55). DDX58 plays a major role in sensing viral infection and in the activation of antiviral responses including the induction of type I interferons and proinflammatory cytokines, and DHX58 acts as a regulator of DDX58 mediated antiviral signaling and can facilitate viral RNA recognition by DDX58 through its ATPase domain (56). It has been reported that DHX58 was highly expressed in macrophages infected with West Nile virus, and is a nonessential but positive regulator to RLR signaling of innate immune defenses (57). These commonly up-regulated proteins may play a role as regulators in polarization and functional characteristic of macrophages.

It could also be predicted that some protein expression differences should certainly existed between the activated macrophages of human and mouse cell models. Of note, differences between human and mouse macrophages have been reported in gene regulation and immunometabolism in response to LPS (one of M1 stimulus), as well as in the immunological responses to TLR4 signaling, of which TLR4 is a LPS receptor (58, 59). Furthermore, IL-4 and IL-13 (M2 stimulus) have overlapping but non-redundant activities on macrophages (5, 60). Our quantitative proteome analysis revealed that both M1 and M2 macrophages had considerable divergences in protein expression between human THP-1 and mouse RAW264.7 cell lines-derived macrophages. For each polarized macrophage, we have identified many proteins that were exclusively up-regulated in human or mouse cell model, even though the specific mechanism still needs further research. It is worth noting that limitation of this study may be the different effects of microenvironment *in vitro* and *in vivo*. Recently, a study showed that pro-inflammatory stimulus (LPS and IFN-γ) did not cause expression change of SOD2 in RAW264.7 macrophages, this is in agreement with our results that SOD2 was only significantly up-regulated in human M1 macrophages. However, the presence of carnosine during pro-inflammatory stimulation caused a significant increase in the gene expression of SOD2 in mouse macrophages. Carnosine is widely distributed in mammalian tissues, which suggests that we must consider the effect of microenvironment to different macrophage models (61).

## Conclusion

In conclusion, by performing a systematically proteomic analysis on polarized macrophages from human THP-1 and mouse RAW264.7 cell lines, we compared the protein expression profiles of different types of polarized macrophages as well as the two macrophage-like cell lines. The study not only identified the commonly changed proteins in M1 or M2 macrophages from human THP-1 and mouse RAW264.7 cells, but also observed considerable differences existed in each type of polarized macrophages between two cell lines. These data provide important reference for new shared biomarker discovery of polarized macrophages in human and mouse, as well as will benefit the preclinical development of therapies for related diseases using the appropriate cell models.

## Data Availability Statement

The mass spectrometry data have been deposited to the ProteomeXchange Consortium (http://proteomecentral.proteomexchange.org) *via* the PRIDE partner repository with the dataset identifier PXD019800.

## Author Contributions

PL and SS designed the experiments. PL and ZH performed experiments with help from RL. CM and BZ performed MS analysis. ZH, JW, YX, PR and DF analyzed the data. ZH, JL and SS wrote the paper with help from all the other co-authors. All authors contributed to the article and approved the submitted version.

## Funding

This work was supported by National Key Research and Development Project (No. 2019YFA09005200), National Natural Science Foundation of China (Nos. 91853123, 81773180 and 21705127), China Postdoctoral Science Foundation (Grant Nos. 2019TQ0260 and 2019M663798), and the General Project of International Science and Technological Cooperation of Shaanxi Province (No. 2019KW-071).

## Conflict of Interest

The authors declare that the research was conducted in the absence of any commercial or financial relationships that could be construed as a potential conflict of interest.

## Abbreviations

M1, classically activated macrophage; M2, alternatively activated macrophages; PMA, phorbol-12-myristate-13-acetate; PBS, phosphate buffered saline; IFN-γ, interferon-γ; LPS, lipopolysaccharide; IL-4, interleukin-4; IL-13, interleukin-13; DTT, dithiothreitol; IAM, iodoacetamide; TFA, trifluoroacetic acid; FA, formic acid; ACN, acetonitrile; HCD, higher-energy collisional dissociation; LFQ, label-free quantitation; PRM, parallel reaction monitoring; FDR, false discovery rate; PSMs, peptide-spectrum matches; GO, Gene Ontology; KEGG, Kyoto Encyclopedia of Genes and Genomes; PPI, protein–protein interaction; PCA, Principal component analysis; TLR4, toll-like receptor 4; ISGs, interferon-stimulated genes.
