# Supplementary material for: Comparative Proteomic Analysis of Polarized Human THP-1 and Mouse RAW264.7 Macrophages
Source: Front Immunol. 2021 Jun 29;12:700009. doi: 10.3389/fimmu.2021.700009 (PMC8276023; doi:10.3389/fimmu.2021.700009)
Supplement: Supplementary file 1 [file DataSheet_1.docx]

Supplementary Material

# Supplementary Figures and Tables:

Fig. S1 Page 2

Fig. S2 Page 3

Fig. S3 Page 4

Table S1: qPCR primer sequences for CMPK2, RSAD2, DDX58, DHX58, and GAPDH from human and mouse.

Table S2: Proteins identified and quantified from the global proteome of the human THP-1 macrophages.

Table S3: Differentially expressed proteins in M1 compared with M0 macrophages from PMA-THP-1s.

Table S4: Differentially expressed proteins in M2 compared with M0 macrophages from PMA-THP-1s.

Table S5: Commonly up-regulated and down-regulated proteins in both M1 and M2 compared with M0 macrophages from PMA-THP-1s.

Table S6: Proteins uniquely up-regulated in M1 polarization compared with M0 and M2 cells from the THP-1 macrophages.

Table S7: Proteins uniquely up-regulated in M2 polarization compared with M0 and M1 cells from the THP-1 macrophages.

Table S8: Up-regulated proteins under polarization conditions for the human THP-1 macrophages.

Table S9: Proteins identified and quantified from the global proteome of the mouse RAW264.7 macrophages.

Table S10: Differentially expressed proteins in M1 compared with M0 macrophages from RAW264.7 cells.

Table S11: Differentially expressed proteins in M2 compared with M0 macrophages from RAW264.7 cells.

Table S12: Commonly up-regulated and down-regulated proteins in both M1 and M2 compared with M0 macrophages from RAW264.7 cells.

Table S13: Proteins uniquely up-regulated under each polarized condition compared with other two phenotypes from the RAW264.7 macrophages.

Table S14: Up-regulated proteins under polarization conditions for the mouse RAW264.7 macrophages.

Table S15: Commonly up-regulated proteins of M1 polarization compared with M0 and M2 macrophages (4FC) in both the THP-1 and RAW264.7 cell models.

Table S16: Commonly up-regulated proteins of M1 polarization compared with M0 and M2 macrophages (2FC) in both the THP-1 and RAW264.7 cell models.

Table S17: Differentially expressed proteins between human THP-1 and mouse RAW264.7 polarized macrophages.

# Supplementary Figures


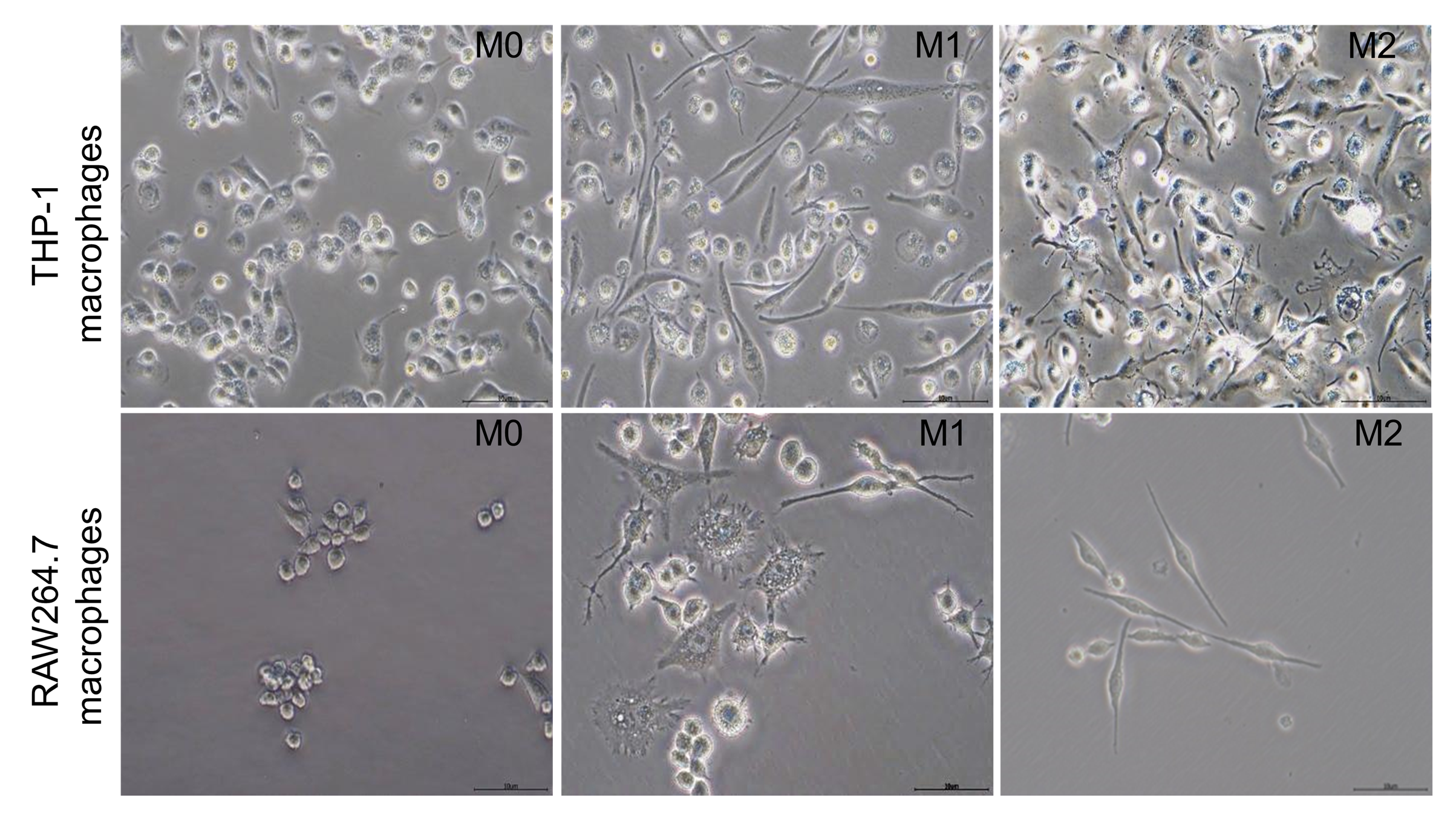


**Fig. S1.** Morphological characteristics of different polarized and unpolarized macrophages from both human THP-1 and mouse RAW264.7 cell models (bar = 10 μm). Polarized macrophages were induced by different stimulus conditions, as described in the Experimental Section and Results sections.


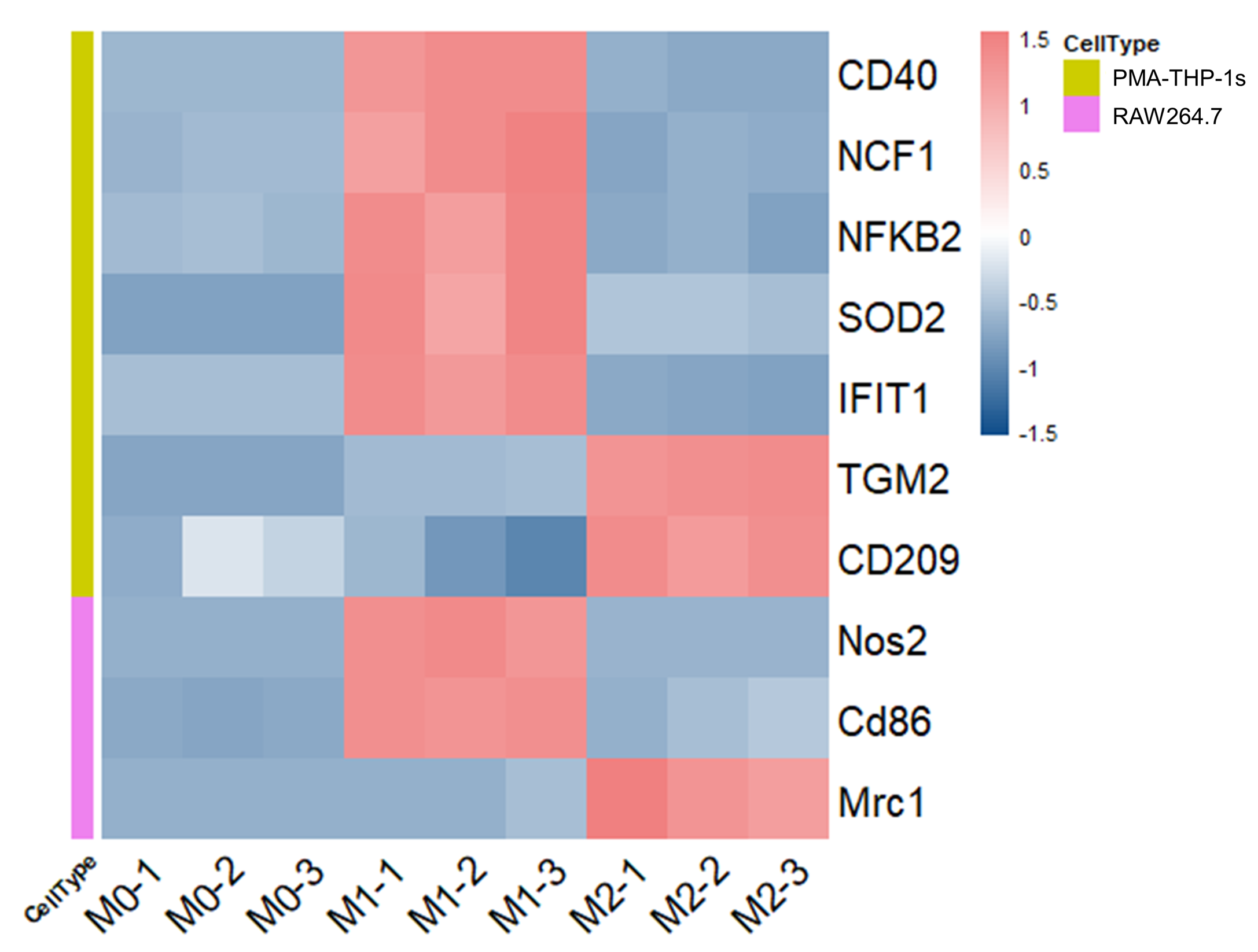


**Fig. S2.** Heatmap showing the expression of the known markers or over-expressed proteins for polarized macrophage (M1and M2) from two cell models in our proteomics. The color bar indicates the Z-score of abundances. In THP-1 cells, the protein CD40, NCF1, NFKB2, SOD2, and IFIT1 for M1; TGM2 and CD209 for M2. In RAW264.7 cells, the Nos2 and Cd86 are M1 markers, Mrc1 being M2 markers.


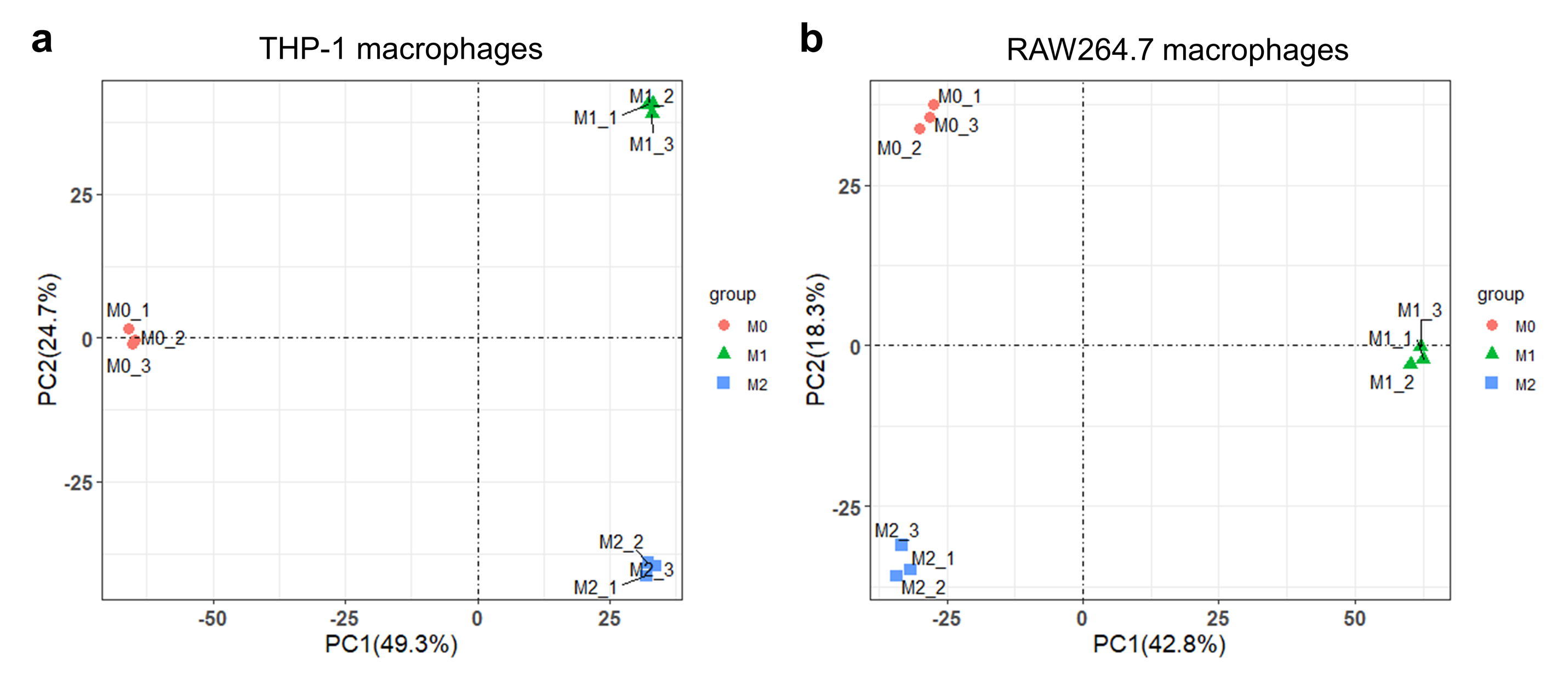


**Fig. S3.** Principal component analysis of quantitative proteins for THP-1 (a) and RAW264.7 macrophages (b). The values of the first and second principal components were showed for three repeats of each phenotype.
